# Reduced Fascial Dehiscence with Combined Small-and-Large Technique Compared to Small-Bite Technique in Emergency Midline Laparotomy: A Retrospective Study

**DOI:** 10.1007/s10029-025-03536-z

**Published:** 2026-01-20

**Authors:** Faruk Koca, Svenja Sliwinski, Konstantin Uttinger, Ekaterina Petrova, Niels Matthes, Armin Wiegering, Tamás Benkö

**Affiliations:** Department of General, Visceral, Transplant and Thoracic Surgery, Goethe University Frankfurt, University Hospital, Frankfurt am Main, Theodor-Stern-Kai 7, 60590 Germany

## Abstract

**Purpose:**

This study aimed to compare the rates of fascial dehiscence after fascial closure using the combined small-and-large technique versus the small-bite technique following emergency laparotomy.

**Methods:**

A retrospective, single-center, observational study was conducted. All patients who underwent emergency midline laparotomy at the University Hospital Frankfurt in from 2022 till 2024 with either small-bite technique were included or combined small-and-large technique, which involves periodic internal reinforcement sutures. Propensity score matching was performed based on preoperative predictors of fascial dehiscence. The rate of fascial dehiscence was compared between the matched groups.

**Results:**

A total of 294 patients were included. The combined small-and-large technique was used for fascial closure in 37 cases (12.6%), while the small-bite technique was used in 257 cases (87.5%). In descriptive statistics, fascial dehiscence was observed in 31 cases (10.5%); two cases (5.4%) in the group with combined small-and-large technique and 29 cases (11.3%) in the group with small-bite technique. After perfoming propensity score matching 29 cases were matched in each group, with one case of fascial dehiscence in the combined small-and-large technique versus 5 cases the small-bite technique group. However, there was no statistically significant difference.

**Conclusion:**

A lower rate of fascial dehiscence may be achieved using the combined small-and-large technique following emergency laparotomy compared to the small-bite technique.

**Supplementary Information:**

The online version contains supplementary material available at 10.1007/s10029-025-03536-z.

## Introduction

Postoperative fascial dehiscence (FD), also known as a “burst abdomen,” is a common complication of laparotomy. It is reported to occur at an increased rate of 8.5% to 45% in emergency procedures [1]. An analysis revealed an elevated in-hospital mortality rate and increased need for prolonged therapeutic interventions among patients who underwent relaparotomy, primarily due to FD [2]. These patients are also reported to have a higher incidence of postoperative complications, resulting in a longer and more complicated hospital stay [2].

There are several techniques for fascial closure, including the small-bite technique, mass or layered closure, reinforced tension-line or interrupted suturing, the Hughes abdominal wall, and the modified Smead-Jones closure technique [3]. The continuous small-bite technique (S-T) with slowly absorbable suture material is recommended for fascial closure during elective midline laparotomies [4]. However, there are currently no recommendations regarding the optimal technique for emergency fascial closure [4]. The aim of this study was to compare the incidence of FD after fascial closure using the S-T versus the combined small-and-large technique following emergency midline laparotomy. This technique involves placing individual sutures at five-centimeter intervals, 15 millimeters from the edges of the wound, to provide periodic internal reinforcement. This is referred to as the combined small-and-large technique (SL-T).

## Materials and methods

This is a retrospective, single-center, observational study. It was approved by the local ethics committee of the Faculty of Medicine at the Goethe University Frankfurt, Germany (reference number: 2024–2205). A screening was conducted on all cases who underwent emergent midline laparotomy at the Department of General, Visceral, Transplant and Thoracic Surgery, University Hospital Frankfurt, from January 2022 to December 2024.

### Patients and screening

Data were retrospectively collected from the electronic patients’ records. Surgical procedures are systematically classified into five distinct categories, from A to E, with emergency operations designated as categories A to D. The categories are as follows: A for immediately life-threatening disorders that require immediate intervention, B for indirectly life-threatening disorders necessitating the allocation of the next available operating room capacity, C for serious or relevant damage to health that is expected to occur within six hours and necessitating surgery within six hours of registration, D for operations that are not significantly life-threatening and can be postponed for up to 24 h with surgery occurring within 24 h of registration, and E for elective procedures and surgeries that take place during normal working hours. All laparotomies in the categories from A to D were selected for further screening.

The S-T involves making tissue bites measuring 5–9 mm from the wound edges to incorporate the aponeurosis. Stitches are placed 5 mm apart to ensure adequate tension distribution. This technique is performed using a slowly absorbable suture material [4] (Monomax Poly − 4-hydroxybutyrate- monofilament, absorbable VIOLET, 150 cm, 0 met. 3,5, HR26, BRAUN, B. Braun Surgical, S.A. Rubi. Spain). This has been standard method since 2019. However, a high rate of FD was observed in emergency laparotomies. Therefore, some surgeons have modified this method by taking large bites individually. We refer to this modified method as the SL-T.

The SL-T involves taking tissue bites measuring 5–9 mm from the wound edges to incorporate the aponeurosis. Stitches are placed 5 mm apart to ensure adequate tension distribution. This technique uses a slowly absorbable suture material [4] (Monomax Poly − 4-hydroxybutyrate- monofilament, absorbable VIOLET, 150 cm, 0 met. 3,5, HR26, BRAUN, B. Braun Surgical, S.A. Rubi. Spain). An additional suture with a single button was applied to tissue bites 15 millimeters from the wound edges for every five centimeters of continuous fascial closure. This was done using a multifilament thread (Novosyn 90/10 Poly (glycolide-co-L-lactide), braided, coated, absorbable VIOLET, 45 cm, 2 met. 5, HR48, BRAUN, B. Braun Surgical, S.A. Rubi. Spain).

### Demographic and clinical characteristics

Age at surgery, sex, presence of pre-existing comorbidities, and medications were recorded. Comorbidities include pulmonary disease (asthma or chronic obstructive pulmonary disease), diabetes mellitus, chronic kidney disease, cardiovascular disease or hypertension, chronic liver disease, active malignancy at the time of surgery, and hematologic disease. Medications include immunosuppressive drugs, anticoagulants, antiplatelet agents, and chemotherapy within twelve months prior to surgery. The American Society of Anesthesiologists (ASA) classification [5] and body mass index (BMI) were calculated. BMI was categorized according to the World Health Organization (WHO) system [6] in kg/m^2^ as follows: <18.5, 18.5–24.9, 25–29.9, 30–34.9, 35–39.9, and ≥ 40.

### Procedure-related parameters

The following diagnoses, for which midline laparotomy was performed, were summarized based on the operation reports: no pathology; paralytic/adhesive ileus or volvulus; hollow organ perforation; mesenteric ischemia; septic or hemorrhagic ischemia; cancer with obstruction or bleeding; incarcerated hernia; complicated diverticulitis; chronic inflammatory bowel disease; anastomotic leak; bleeding; and other injuries, including traumatic injuries.

The following surgical procedures were defined: only exploration or adhesiolysis, suture of a hollow organ perforation, small bowel resection, colorectal resection with anastomosis, bowel resection with loop ostomy, bowel resection with end ostomy, and other procedures. Concurrent gastrointestinal procedures were noted if a gastric or bowel resection was performed.

FD was diagnosed through local wound examination and bedside evaluation. Revision surgery was indicated because the fascial suture was dehiscent and exposed the intra-abdominal organs. Cases in which a revision relaparotomy was primarily necessary due to fascial dehiscence were counted as cases of FD. It was documented according to International Statistical Classification of Diseases and Related Health Problems (ICD) 10th Revision with the ICD code T81.3 [7], and the surgical procedure was designated as abdominal wall closure for burst abdomen with operation and procedure codes (OPS) code 5–545.0.0 in accordance with the German Federal Institute for Drugs and Medical Devices [8]. During the revision surgery, FD was recorded as the main diagnosis.

Postoperative complications were classified as major complications ≥ III according to the Clavien-Dindo classification (CDC) [9]. The following were documented: computed tomography (CT) scans, magnetic resonance imaging (MRI), preoperative values of laboratory parameters, including white blood cell count (/nL), hemoglobin level (g/dL), creatinine level (mg/dL), and lactate level (mg/dL). Additionally, histopathological findings were recorded.

The follow-up focused on the length of the hospital stay, measured in days. The length of stay in the intensive and intermediate care units was also recorded in days. The timepoint of FD diagnosis, as well as the timepoint of the last medical assessment after surgery (including outpatient visits), was calculated in days.

#### Statistical analysis

Statistical analysis was performed using IBM SPSS version 29.0.0.2 software. Descriptive statistics with median and percentage of total for binary and categorical parameters as well as median and interquartile range (IQR) for continuous parameters were calculated.

Chi-squared tests were used to compare the SL-T with the S-T for the following parameters: sex, performing CT or MRI, surgical procedure involving gastric or bowel resection, and occurrence of major complications (CDC ≥ 3). The Mann-Whitney U test was used to compare the length of follow-up, the length of hospital stay, and the length of stay in the intensive and intermediate care units, age at the time of surgery, white blood cells (/nL), hemoglobin (g/L), lactate (mg/dL), and creatinine (mg/dL).

Propensity score matching with the 1:1 nearest method was performed using univariable binary logistic regression to establish a propensity score with probabilities based on known preoperative predictors of fascial dehiscence or incisional hernia [10]. These predictors were BMI, ASA status, pumonary disease, hypertension, active cancer, chemotherapy, chronic liver disease, use of antiplatelet or anticoagulants, concurrent gastrointestinal procedures (e.g., gastric or bowel resection), and acute peritonitis [10]. Fisher’s exact test was used to compare the groups regarding FD.

## Results

A total of 834 emergency laparotomy cases were screened. Of those, 78 patients died within ten days of hospitalization, and one patient was transferred to another hospital immediately after surgery. Therefore, 79 patients were excluded due to insufficient follow-up regarding FD. 16 cases involving multiorgan procurement could not be included.

A total of 248 cases did not have a midline incision and were therefore excluded. Of these, 65 had a reversed L-incision for a liver transplant, 118 had a Gibson or hockey stick incision for a kidney transplant, 38 had a transverse upper abdominal laparotomy, 22 had a subcostal, one had a Mercedes star, two had a Pfannenstiel, and one had a flank incision.

**Fig. 1:**
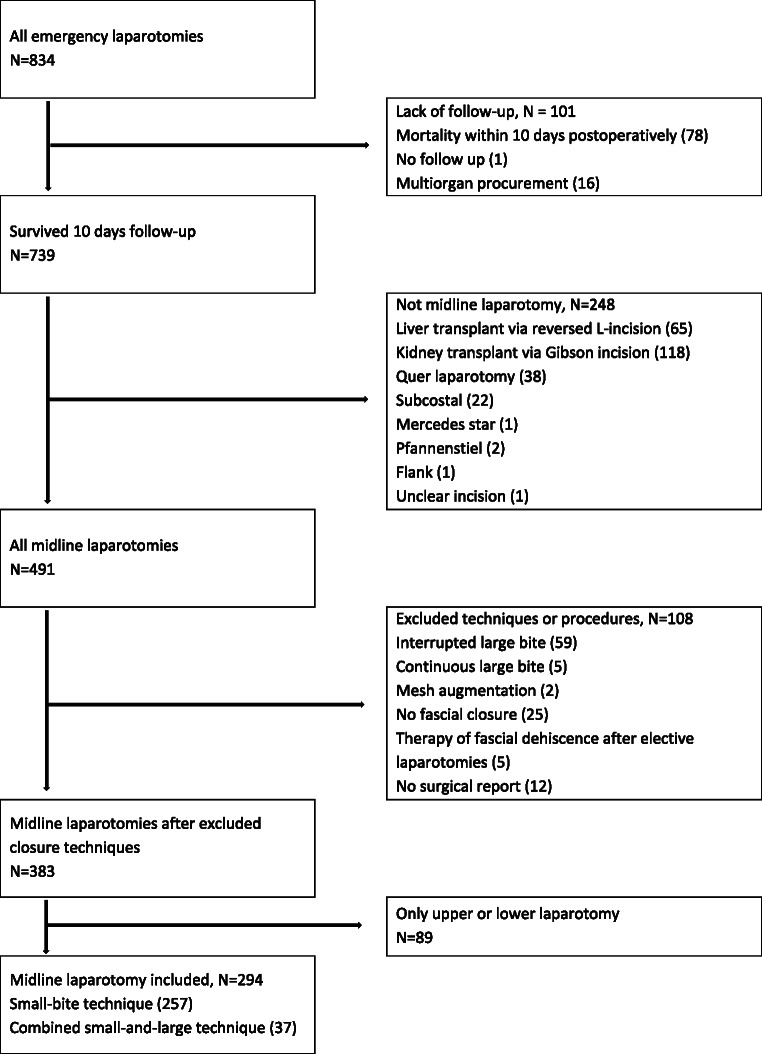
Flowchart of the screening process

As this study compared two fascial closure techniques (S-T versus SL-T), cases involving other techniques with 108 cases were excluded. Of these, 59 had an interrupted large-bite technique, five had a continuous large-bite technique, two had mesh augmentation, and 25 had an abdominal aperture due to a lack of fascial closure. Five cases involving fascial closure due to dehiscence as part of the treatment following elective laparotomy were excluded, as were 12 cases for which no surgical report was available. 89 cases involving only an upper or lower laparotomy were excluded because suture length is considered a predictive factor for FD [10]. An overview is presented in the flowchart of the screening process (Fig. 1).

### Demographic and procedure-related characteristics

A total of 294 cases were included in the study. The median age of the patients at the time of the surgery was 63 with IQR of 22 years. Of the cases, 171 (58.2%) were male patients and 123 (41.8%) were female patients. 268 cases (91.2%) underwent preoperative imaging. The median white blood cell count was 11/nL (IQR 10/nL), the median hemoglobin level was 10.1 g/dL (IQR 3.8 g/dL), the median lactate level was 15 mg/dL (IQR 17 mg/dL), and the median creatinine level was 1.1 mg/dL (IQR 0.9 mg/dL).

Nine cases (3.1%) underwent laparotomy to rule out a suspected diagnosis, which revealed no pathology. Sixty-six cases (22.4%) involved paralytic or adhesive ileus, volvulus, or both; 46 cases (15.6%) involved perforation of a hollow organ; 19 cases (6.5%) involved mesenteric ischemia; 19 cases (6.5%) involved septic or hemorrhagic ischemia; 39 cases (13.3%) involved cancer with obstruction or bleeding; and nine cases (3.1%) involved incarcerated hernias; 20 cases (6.8%) involved diverticulitis; 13 cases (4.4%) involved chronic inflammatory bowel disease; 18 cases (6.1%) involved anastomotic leaks after bowel resections; 19 cases (6.5%) involved bleeding from intra-abdominal organs; and 22 cases (7.5%) involved midline laparotomies due to other diagnoses, including abdominal trauma.

A gastric or bowel resection was performed in 169 (57.5%) cases, of those 38 cases (12.9%) were small bowel resections with anastomosis; 36 cases (12.2%) were colorectal resections with anastomosis; 26 cases (8.8%) were bowel resections with a loop ostomy; and 66 cases (22.4%) were bowel resections with an end ostomy. Sixty-seven cases (22.8%) involved exploration or adhesiolysis only, 20 cases (6.8%) involved suturing a perforation, and 40 cases (13.6%) involved other procedures, such as controlling bleeding or splenectomy. 37 cases (12.6%) had fascial closure in SL-T and 257 cases (87.4%) in S-T.

The median hospital stay was 13 days (IQR, 12 days) and the median time to the final evaluation was 97 days (IQR, 253 days). The median time from surgery to FD detection was eight days (IQR, 10 days). There were 89 cases (30.3%) with major complications (CDC ≥ 3). No statistically significant differences were found in the demographic and clinical characteristics between patients with SL-T and those with S-T (Table 1). 

**Table 1:** Comparison of the demographic and clinical characteristics of patients who underwent midline emergent laparotomy using the combined small-and-large versus small-bite technique

*N*(%)/median (IQR)	Total *N* = 294	Combined small-and-large technique *n* = 37	Small-bite technique *n* = 257	*P*
Age at the time of surgery [years]	63 (22)	63 (30)	63 (21)	0.674
Sex				0.379
Male	171 (58.2)	19 (51.4)	152 (59.1)	
Female	123 (41.8)	18 (48.6)	105 (40.9)	
Imaging and laboratory				
CT or MRI	268 (91.2)	32 (86.5)	236 (91.8)	0.862
White blood cells (/nL)	11 (10)	11 (10)	12 (12)	0.675
Hemoglobin (g/dL)	10.1 (3.8)	10.1 (3.7)	12 (5.1)	0.562
Lactate (mg/dL)	15 (17)	14 (15)	29 (39)	0.079
Creatinine (mg/dL)	1.1 (0.9)	1 (0.9)	1.3 (1.4)	0.793
Diagnoses				0.950
No pathology	9 (3.1)	1 (2.7)	8 (3.1)	
Paralytic/adhesive ileus or volvulus	66 (22.4)	7 (18.9)	59 (23)	
Viscous organ perforation	46 (15.6)	5 (13.5)	41 (16)	
Mesenteric ischemia	19 (6.5)	3 (8.1)	16 (6.2)	
Septic/hemorrhagic ischemia	13 (4.4)	3 (8.1)	10 (3.9)	
Cancer with obstruction or bleeding	39 (13.3)	4 (10.8)	35 (13.6)	
Incarcerated Hernia	9 (3.1)	1 (2.7)	8 (3.1)	
Diverticulitis	20 (6.8)	3 (8.1)	17 (6.6)	
Chronic inflammatory bowel disease	13 (4.4)	0	13 (5.1)	
Anastomotic leak	18 (6.1)	3 (8.1)	15 (5.8)	
Bleeding	19 (6.5)	3 (8.1)	16 (6.2)	
Other	22 (7.5)	3 (8.1)	19 (7.4)	
Surgical procedure				
Exploration/adhesiolyse	67 (22.8)	9 (24.3)	58 (22.3)	
Suture of perforation	20 (6.8)	1 (2.7)	19 (7.4)	
Small bowel resection	38 (12.9)	3 (8.1)	35 (13.6)	
Colorectal resection with anastomosis	36 (12.2)	5 (13.5)	31 (12.1)	
Bowel resection with loop ostomy	26 (8.8)	5 (13.5)	21 (8.2)	
Bowel resection with end ostomy	66 (22.4)	7 (18.9)	59 (23)	
Other	40 (13.6)	6 (16.2)	34 (13.2)	
Definitive closure after damage control surgery	26 (8.8)	2 (5.4)	24 (9.3)	0.750
Hospital stay	13 (12)	13 (12)	12 (20)	0.723
Intensive care unit-stay	0 (3)	0 (2)	0 (5)	0.164
Intermediate care unit-stay	1 (3)	1 (3)	0 (4)	0.949
Major complications CDC ≥ 3	89 (30.3)	8 (21.6)	81 (31.5)	0.290
Timepoint of fascial deshiscence diagnosis (d)	10 (8)	14.5 (n/a)	8 (7)	0.243
Follow-up (d)	97 (253)	108 (264)	58 (222)	0.237

*D* days, *N *number, *IQR* interquartile range, *CDC* Clavien-Dindo classification, *CT* computed tomography, *MRI* magnetic resonance imaging

### Predictors of fascial dehiscence

The ASA classification was I oder II in 48 cases (16.3%) and ≥ III in 242 cases (82.3%), with four cases (1.4%) not documented. The median BMI was 24.8 (IQR, 7.6) kg/m². The median BMI was higher in the SL-T group at 29 (IQR, 9.7) kg/m² than in the S-T group at 24.3 (IQR, 6.7) kg/m² (*p* < 0.001). On the other hand, the BMI could not be determined for 41 cases (13.9%).

In terms of comorbidities, there were no statistically significant differences between the two groups. The following comorbidities were reported: pulmonary disease (17.3%), diabetes mellitus (15.3%), chronic kidney disease (13.9%), cardiovascular disease or hypertension (67%), chronic liver disease (6.1%), active malignancy (34.7%), and hematologic disease (5.1%).

17.3% of cases were taking anticoagulants, 24.5% were taking antiplatelet agents as long-term medication, 6.4% were taking immunosuppressants, and 11.2% had undergone chemotherapy in the previous 12 months.

Of the cases, 132 (44.9%) were classified as peritonitis based on surgical findings and 66 cases (22.4%) presented with shock. There was no significant difference between the two groups. A detailed overview of the FD predictors is provided in Table 2.

**Table 2:** Comparison of predictors and rates of fascial dehiscence following emergent midline laparotomy using the combined small-and-large technique versus the small-bite technique, including propensity score matching

					After propensity score matching			
N(%)/median (IQR)	Total *N* = 294	Combined small-and-large technique *N* = 37	Small bite technique *N* = 257	p	Total *N* = 58	Combined small-and-large technique *N* = 29	Small bite technique *N* = 29	p
**ASA**				1				1
< III	48 (16.3)	6 (16.2)	42 (16.3)		13 (22.4)	6 (20.7)	7 (24.1)	
≥III	242 (82.3)	30 (81.1)	212 (82.5)		45 (77.6)	23 (79.3)	22 (75.9)	
missing	4 (1.4)	1 (2.7)	3 (1.2)					
Body mass index (kg/m2)	24.8 (7.6)	29 (9.7)	24.3 (6.7)	**< 0.001**				0.348
<18.5	16 (5.4)	1 (2.7)	15 (5.8)		2 (3.4)	1 (3.4)	1 (3.4)	
18.5–24.9	115 (39.1)	8 (21.6)	107 (41.6)		16 (27.6)	6 (20.7)	10 (34.5)	
25–29.9.9	66 (22.4)	9 (24.3)	57 (22.2)		18 (31)	9 (31)	9 (31)	
30–34.9.9	33 (11.2)	9 (24.3)	24 (9.3)		11 (19)	8 (27.6)	3 (10.3)	
35–39.9.9	17 (5.8)	5 (13.5)	12 (4.7)		7 (8.6)	3 (10.3)	4 (13.8)	
≥40	6 (2)	2 (5.4)	4 (1.6)		4 (6.9)	2 (6.9)	2 (6.9)	
missing	41 (13.9)	3 (8.1)	38 (14.8)					
Comorbidities/medication								
Pulmonary disease	51 (17.3)	4 (10.8)	47 (18.3)	0.354	5 (8.6)	4 (13.8)	1 (3.4)	0.352
Diabetes mellitus	45 (15.3)	7 (18.9)	38 (14.8)	0.474	10 (17.2)	4 (13.8)	6 (20.7)	0.730
Chronic kidney disease	41 (13.9)	7 (18.9)	34 (13.2)	0.322	9 (15.5)	5 (17.2)	4 (13.8)	1
Cardiovascular disease or hypertension	197 (67)	24 (64.9)	173 (67.3)	0.713	36 (62.1)	18 (62.1)	18 (62.1)	1
Chronic liver disease	18 (6.1)	2 (5.4)	16 (6.2)	1	4 (6.9)	2 (6.9)	2 (6.9)	1
Active malignancy	102 (34.7)	13 (35.1)	89 (34.6)	0.861	23 (39.7)	11 (37.9)	12 (41.4)	1
Hematologic disease	15 (5.1)	3 (8.1)	12 (4.7)	0.353	5 (8.6)	3 (10.3)	2 (6.9)	1
Immunosuppressive drugs	19 (6.4)	1 (2.7)	18 (7)	0.485	1 (1.7)	1 (3.4)	0	1
Chemotherapy	33 (11.2)	5 (13.5)	28 (10.9)	0.586	10 (17.2)	4 (13.8)	6 (20.7)	0.730
Anticoagulants	52 (17.3)	6 (16.2)	46 (17.9)	1	8 (13.8)	4 (13.8)	4 (13.8)	1
Antiplatelet agents	72 (24.5)	6 (16.2)	66 (25.7)	0.303	9 (15.5)	5 (17.2)	4 (13.8)	1
Peritonitis	132 (44.9)	18 (48.6)	114 (44.4)	0.833	29 (50)	16 (55.2)	13 (44.8)	0.600
Shock	66 (22.4)	13 (35.1)	53 (20.6)	0.062	13 (22.4)	9 (31)	4 (13.8)	0.207
Gastric or bowel resection	169 (57.5)	20 (54.1)	149 (58)	0.723	27 (46.6)	16 (55.2)	11 (37.9)	0.292
Fascial dehiscence	31 (10.5)	2 (5.4)	29 (11.3)	0.395	6 (10.3)	1 (3.4)	5 (17.2)	0.097

*ASA* American Society of Anesthesiologists, IQR-interquartile range

### Primary outcome including prospensity score analysis

FD occurred in 31 cases (10.5%). It occurred in 29 (11.3%) cases in the S-T group. This percentage is higher than the 5.4% observed in the SL-T group.

Due to differences in size between the two groups and the absence of certain predictor values, both groups were matched using a propensity score. This method was based on predictors of FD [10], 29 cases were identified in each group. The two groups were matched based on the closest probabilities with nearest 1:1 method. The standard deviation (SD) of the logit values of the propensity scores was multiplied by 0.2. Then, the caliper width was calculated to be 0.219544. The standardized mean differences before and after matching are provided in Supplementary Table 1. Following propensity score matching, FD occurred in 3.4% of cases in the SL-T group and in 17.2% of cases in the S-T group. Thus, the SL-T group exhibited a lower FD rate than the S-T group. A detailed overview of the primary outcome „FD“ is provided in Table 2.

## Discussion

A significant proportion of patients undergoing open surgery experience FD after surgery. This results in increased costs, a longer hospital stay, the need for additional interventions, and complications [11]. The SL-T was used for fascial closure in 37 cases, while the S-T was used in 257 cases. According to the descriptive statistics, FD occurred at a rate of 10.5%, with rates of 5.4% and 11.3% in the SL-T and S-T groups, respectively. After propensity score matching, 29 cases were matched in each group. There was one case of FD in the SL-T group and five in the S-T group. However, there was no statistically significant difference.

The small-bite technique in combination with the single button technique has recently been described, in which the single-button sutures are spaced 2 cm apart [12]; in the method of the current analysis (SL-T), they are spaced 5 cm apart. Using a shorter distance in the large-bite technique may increase trauma to the fascia [13] and also negatively impact the incidence of FD. Regarding this issue, combining the single-button technique with a distance of 5 cm, as defined in the SL-T, may be an alternative solution.

The rate of FD following open surgery is higher in emergency settings. The rate is 5–10% for patients with definitive laparotomy and 13–50% for those undergoing damage control surgery [14]. The FD rate was 5.4% in the group that used the SL-T including patients in severe shock and those who underwent second-look closure after damage control surgery. This rate is low compared to those reported in the literature, where the incidence of FD following emergency laparotomy in the overall cohort is 10.5% in this study. This rate is comparable to those reported in the literature. This means that one out of every ten patients who underwent midline emergent laparotomy required a second abdominal operation under general anesthesia solely due to FD. This issue is considered a major problem, and an innovation like using the SL-T is believed necessary for reducing the FD rate.

Outpatient follow-up assessments were conducted, but important long-term outcomes, such as incisional hernias, could not be analyzed due to the small size of the cohorts. Due to the retrospective nature of the study, there was no standardized follow-up duration.

### Limitations

Different surgical procedures, intra-abdominal infections, and duration of postoperative ventilation in critically ill patients can significantly impact FD. Although propensity score matching was performed on many preoperative predictors, not all parameters were evaluated. The severity of peritonitis and microbial contamination of the fascia were not classified, which also impacts FD directly. The present study has two additional constraints: the lack of an assessment of the peritonitis index and the inability to record the degree to which the surgeons participated (e.g., resident versus consultant). Although only upper or lower midline laparotomies were excluded, the incision diameter was not recorded. The median follow-up period was only 97 days, and the study focused solely on burst abdomen. Incisional hernias were not examined.

Its retrospective nature and small cohort size of only three years are key limitations. This SL-T is not clearly described in the literature and has not been adequately studied. Therefore, multicenter, prospective studies involving larger cohorts and longer follow-up periods are necessary to confirm its advantages.

## Conclusion

A lower rate of fascial dehiscence may be achieved by using the combined small-and-large technique for fascial closure than by using the small-bite technique for emergency midline laparotomy.

## Supplementary Information

Below is the link to the electronic supplementary material.

Supplementary Material 1
