# Supplementary material for: Reduced Fascial Dehiscence with Combined Small-and-Large Technique Compared to Small-Bite Technique in Emergency Midline Laparotomy: A Retrospective Study
Source: Hernia. 2026 Jan 20;30(1):73. doi: 10.1007/s10029-025-03536-z (PMC12819546; doi:10.1007/s10029-025-03536-z)
Supplement: Supplementary file 1 — Supplementary Material 1 [file 10029_2025_3536_MOESM1_ESM.docx]

| Supplementary Table 1 Comparison of the standardized mean differences of the fascial dehiscence predictors following an emergent midline laparotomy using the combined small-and-large technique versus the small-bite technique, before and after propensity score matching. | | | |
| --- | --- | --- | --- |
| **Before propensity score matching,** Total N=294  Combined small-and-large technique N=37, Small bite technique N=257 | |  | **After propensity score matching,** Total N=58  Combined small-and-large technique N=29, Small bite technique N=29 |
|  | Cohen´s d |  | Cohen´s d |
| **ASA**≥III versus <III | 0.373 |  | 0.424 |
| **Body mass index** (kg/m2) according to WHO classification | **1.094** |  | 1.290 |
| **Comorbidities/medication** |  |  |  |
| Pulmonary disease | 0.380 |  | 0.281 |
| Diabetes mellitus | 0.362 |  | 0.383 |
| Chronic kidney disease | 0.348 |  | 0.368 |
| Cardiovascular disease or hypertension | 0.471 |  | 0.494 |
| Chronic liver disease | 0.241 |  | 0.258 |
| Active malignancy | 0.477 |  | 0.498 |
| Hematologic disease | 0.227 |  | 0.285 |
| Immunosuppressive drugs | 0.247 |  | 0.131 |
| Chemotherapy | 0.318 |  | 0.383 |
| Anticoagulants | 0.384 |  | 0.351 |
| Antiplatelet agents | 0.432 |  | 0.368 |
| **Peritonitis** | 0.540 |  | 0.506 |
| **Shock** | 0.419 |  | 0.415 |
| **Gastric or bowel resection** | 0.496 |  | 0.500 |
| **Fascial dehiscence** | 0.308 |  | 0.302 |
| ASA- American Society of Anesthesiologists, WHO- World Health Organization | | | |
